# Intracranial Flow Velocity Quantification Using Non-Contrast Four-Dimensional Flow MRI: A Prospective Comparative Study with Transcranial Doppler Ultrasound

**DOI:** 10.3390/diagnostics12010023

**Published:** 2021-12-23

**Authors:** Sam-Yeol Ha, Yeonah Kang, Ho-Joon Lee, Moonjung Hwang, Jiyeon Baik, Seongho Park

**Affiliations:** 1 Department of Neurology, Haeundae Paik Hospital, Inje University College of Medicine, Busan 48108, Korea; samyeolha@gmail.com (S.-Y.H.); risepsh@gmail.com (S.P.); 2 Department of Neurology, Chung-Ang University Hospital, Chung-Ang University College of Medicine, Seoul 06973, Korea; 3 Department of Radiology, Haeundae Paik Hospital, Inje University College of Medicine, Busan 48108, Korea; hojoon.lee@paik.ac.kr (H.-J.L.); jbaik6@gmail.com (J.B.); 4 GE Healthcare Korea, Seoul 04637, Korea; moonjung.hwang@ge.com; 5 Department of Radiology, Good Gang-An Hospital, Busan 48265, Korea

**Keywords:** blood flow velocity, MRI angiography, transcranial Doppler sonography

## Abstract

Four-dimensional (4D) flow magnetic resonance imaging (MRI) allows three-dimensional velocity encoding to measure blood flow in a single scan, regardless of the intracranial artery direction. We compared blood flow velocity quantification by non-contrast 4D flow MRI and by transcranial Doppler ultrasound (TCD), the most widely used modality for measuring velocity. Twenty-two patients underwent both TCD and non-contrast 4D flow MRI. The mean time interval between TCD and non-contrast 4D flow MRI was 0.7 days. Subsegmental velocities were measured bilaterally in the middle cerebral and basilar arteries using TCD and non-contrast 4D flow MRI. Intracranial velocity measurements using TCD and non-contrast 4D flow MRI demonstrated a strong correlation in the bilateral M1, especially at the proximal segment (right r = 0.74, left r = 0.78; all *p* < 0.001). Mean velocities acquired with 4D flow MRI were approximately 8 to 10% lower than those acquired with TCD according to the location of M1. Intracranial arterial flow measurements estimated using non-contrast 4D flow MRI and TCD showed strong correlation. 4D flow MRI enables simultaneous assessment of vascular morphology and quantitative hemodynamic measurement, providing three-dimensional blood flow visualization. 4D flow MRI is a clinically useful sequence with a promising role in cerebrovascular disease.

## 1. Introduction

Intracranial velocity measurement is clinically important in cerebrovascular conditions such as ischemic stroke, vasospasm, and post-bypass surgery, and it provides valuable hemodynamic information in cases of aneurysm and neurodegenerative disease [1,2,3,4,5]. Transcranial Doppler ultrasound (TCD), which transmits ultrasound waves through the skull and receives reflecting waves from moving red blood cells, has been widely used for noninvasive and real-time measurement of intracranial arteries [6]. However, TCD is limited in insonation angle correction for vascular direction as well as in accurate localization of vascular segments [7,8]. It also has high operator dependency, which results in poor vascular evaluation reproducibility [6,8]. Moreover, insufficient temporal bone window penetration often renders the exam impossible [6,8].

Standard two-dimensional phase-contrast MRI has gained attention as an alternative to TCD that can provide both vascular morphology information and quantitative measurements [9]. However, it is unidirectional velocity encoding that requires setting a target lesion before scanning [10]. Recent developments of MR hardware and technique have enabled acquisition of volumetric time-resolved change over the cardiac cycle known as four-dimensional (4D) flow MRI; in other words, three-dimensional (3D) phase-contrast MRI plus time [10,11,12,13]. With a reasonable scan time, 4D flow MRI allows three-directional velocity encoding by a single scan to measure blood flow velocity regardless of intracranial artery direction [11]. Four-dimensional flow MRI offers a comprehensive hemodynamic assessment via several visualization options, such as color-coded 3D multiplanar reformations, vector graph, streamline, pathline, and other 3D particle trace techniques [12,13]. Although 4D flow MRI is chiefly applied to cardiovascular diseases including congenital heart disease and valvular disease, several studies have reported promising outcomes for cerebral artery aneurysm and arteriovenous malformation [13,14,15,16,17,18].

The 4D flow MRI can be performed without a contrast agent. However, use of a contrast agent provides a significantly improved signal-to-noise ratio, particularly for the smaller branches [19]. Therefore, many published intracranial studies using 4D flow MRI have used a contrast agent, which is advantageous in that it enhances the visibility of small vessels such as the intracranial arteries [19,20,21,22]. Conversely, a previous study, which evaluated the influence of contrast agent on flow velocity measurement in intracranial arteries, recommended performing phase-contrast MRI before contrast administration for accurate velocity measurement in small vessels in order to reduce background signal averaging effects [23].

In this study, we investigated the velocity information of intracranial arteries using 4D flow MRI without contrast. The aim of this study was to compare intracranial artery velocity measurements between non-contrast 4D flow MRI and TCD, which is the traditional and foremost reference standard, to validate the feasibility of 4D flow MRI to provide reliable arterial flow velocity quantification.

## 2. Materials and Methods

### 2.1. Patients

This prospective study was approved by the institutional review board of our institution, and informed consent was obtained from all patients. From December 2019 to March 2020, 22 consecutive patients (age 49.8 ± 13.8 years, 9 women) who visited our neurology department were enrolled in this study. The inclusion criteria were as follows: (a) adults able to undergo MRI scanning; and (b) no known history of cerebrovascular disease. The exclusion criteria were as follows: (a) bilateral poor temporal window on TCD; and (b) poor image quality of 4D-flow MRI. Among the included patients, 20 patients had no significant lesion in the circle of Willis, and 2 patients had mild focal stenosis in the M1 and basilar artery, respectively.

The mean time interval between TCD examination and 4D flow MRI was 0.7 ± 1.6 days. The 2 patients with poor left temporal windows were included in the analysis for the right middle cerebral artery (MCA) and basilar artery.

### 2.2. Magnetic Resonance Imaging

All examinations were performed on a 3T MR imaging system (Signa Architect, GE Healthcare, Milwaukee, WI, USA) with a 48-channel head coil. 4D flow MRI was performed with non-contrast technique. MRI sequences included intracranial time of flight MRA and 4D flow MRI. Imaging parameters for 4D flow MRI were the following: FOV = 200 × 200 mm^2^, TR/TE = 5.5/2.9 ms, VENC = 80–100 cm/s, flip angle = 8°, bandwidth = 50 kHz, matrix = 200 × 200, number of slabs = 80, spatial resolution for the composite image = 0.5 × 0.5 × 1 cm^3^. Retrospective cardiac gating with electrocardiogram was acquired for 4D flow MRI. The 20 time-frames per heartbeat resulted in a temporal resolution that varied from 42 to 56 ms depending on the patient’s heart rate. Consequently, total acquisition times were between 10 and 12 min.

### 2.3. Transcranial Ultrasound Examination

A neurologist (S.Y.H, 20 years of experience) performed the TCD examinations. The TCD studies were acquired on a PMD 150 Doppler (Spencer Technology, Seattle, DC, USA) using a 2-MHz PW transducer. The M1 segments of MCA were insonated through a transtemporal window. The distal M1 segments were detected first at superficial depth. Sequentially, the mid-M1 segments were measured and the proximal M1 segments were obtained until a flow reversal of the anterior cerebral artery was visualized on the window. For the basilar artery, a transforaminal window was utilized. To locate the basilar artery with TCD, the higher flow signals were directed away from the probe, then putative vertebral artery flow around the depth of 70 mm through the window was chosen.

### 2.4. MR Flow Velocity Analysis

**Figure 1:**
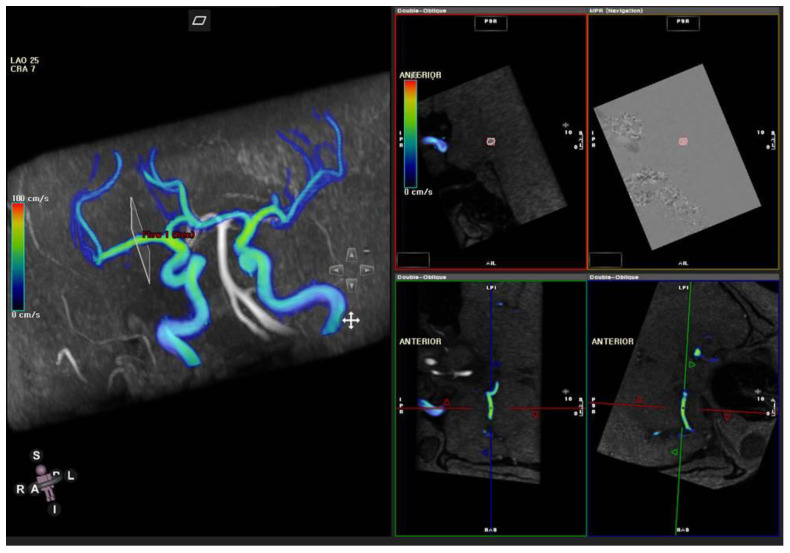
A representative image to set an analysis plane at right mid-M1 segment perpendicular to the vessel direction using commercial software.

Automated post-processing of 4D flow MRI datasets was performed using commercial software (CVI 42, Circle Cardiovascular, Calgary, AB, Canada) and included offset correction and phase unwrapping for antialiasing. Three analysis planes for bilateral M1s and one analysis plane for the basilar artery were positioned at the location measured with TCD using distance correlation. Subsequently, the analysis planes were adjusted to a plane perpendicular to the vessel direction (Figure 1). The maximum velocity over the cardiac cycle was designated as the peak systolic velocity (PSV), and the minimum velocity was designated as the end-diastolic velocity (EDV). Mean velocity (MV) was derived according to the same calculation method in TCD as follows: MV = (PSV + 2 × EDV)/3.

### 2.5. Statistical Analysis

Pearson’s correlations were acquired between velocity measurements of TCD and 4D flow MRI. Bland-Altman plots were generated comparing TCD and 4D flow MRI. The differences of bilateral MCA flow velocities between TCD and 4D flow MRI were compared with a *t*-test. A *p*  <  0.05 was considered statistically significant. All statistical analyses were performed using R for Windows version 3.0.2.

## 3. Results

### 3.1. Flow Velocity Comparison between TCD and 4D Flow MRI

**Figure 2:**
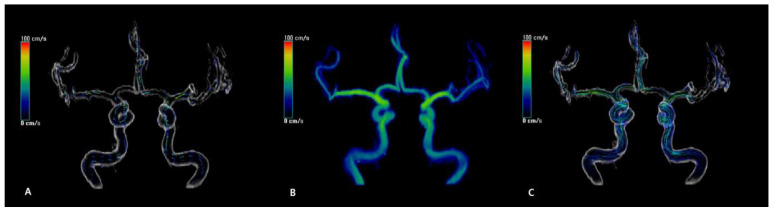
Static captures of four-dimensional flow MRI. Anterior circulation of a 39-year-old woman with normal intracranial arteries. (**A**) Color-coded three-dimensional (3D) rendering shows velocity vectors, which visualize the direction of blood flow; (**B**) Color-coded 3D rendering of flow velocity, which displays velocity changes according to the cardiac cycle; (**C**) Color-coded 3D rendering with streamlines, which represent the path of particles if the particles are released into the velocity field while the field is kept constant.

Figure 2 and the Supplementary Materials show representative cases of velocity, path line, and vector plots of the circle of Willis. The MV (±SD) measured by TCD in distal M1 was 46.59 ± 10.42 cm/s, PSV was 66.76 ± 14.87 cm/s, and EDV was 33.04 ± 8.29 cm/s. The MV (±SD) measured by 4D flow MRI in distal M1 was 41.85 ± 10.44 cm/s, PSV was 59.31 ± 15.59 cm/s, and EDV was 32.86 ± 8.67 cm/s. In mid M1, the MV (±SD) measured by TCD was 55.26 ± 11.75 cm/s, PSV was 78.23 ± 15.63 cm/s, and EDV was 39.76 ± 9.34 cm/s. Using 4D flow MRI, the MV (±SD) was 50.18 ± 10.37 cm/s, PSV was 71.17 ± 15.03 cm/s, and EDV was 39.69 ± 8.99 cm/s. In proximal M1, the MV (±SD) measured by TCD was 55.65 ± 13.00 cm/s, PSV was 79.78 ± 17.70 cm/s, and EDV was 39.48 ± 10.29 cm/s. Using 4D flow MRI, the MV (±SD) was 51.64 ± 9.78 cm/s, PSV was 73.76 ± 13.27 cm/s, and EDV was 40.58 ± 8.75 cm/s in proximal M1.

**Figure 3:**
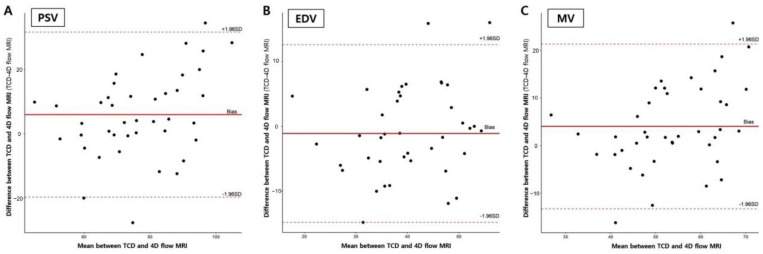
Bland-Altman plots of four-dimensional flow MRI compared with transcranial Doppler ultrasound in the proximal M1 segments. (**A**) Peak systolic velocity; (**B**) End diastolic velocity; (**C**) Mean velocity.

**Figure 4:**
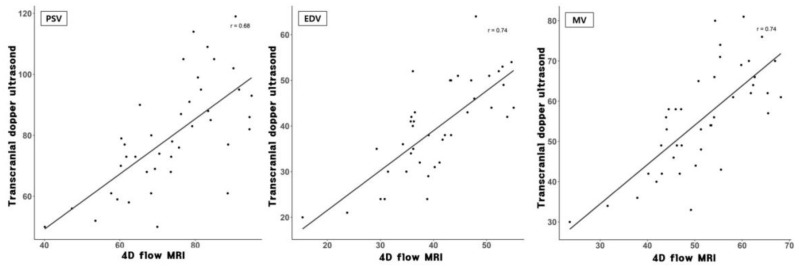
Correlation plots between four-dimensional flow MRI and transcranial Doppler ultrasound in the proximal M1.

The velocity values from all vascular locations (right and left M1 and basilar artery) and correlation coefficients between the two modalities are summarized in Table 1. Bland-Altman analysis for MV in the proximal M1 using 4D flow MRI compared with that using TCD showed that 40 of 42 points were within 1.96 SDs, with a bias of 4.01, and 1.96 SD limits of agreement of −12.9 and 21.1. This implied that velocities determined using 4D flow MRI were approximately 8% lower than those determined using TCD, and this trend was in line with the mid and distal M1 segments. Bland-Altman analyses of 4D flow MRI compared with TCD in proximal M1 are presented in Figure 3. The 4D flow MRI and TCD were well correlated, especially in the proximal M1 with r = 0.74 and r = 0.78 for the right and left sides, respectively (all *p* < 0.001). Figure 4 shows correlation plots between 4D flow MRI and TCD.

### 3.2. Differences of Bilateral MCA Flow Velocities

The MCA velocity data obtained from 20 patients with sufficient bilateral temporal windows were analyzed. Although the velocity difference of bilateral M1 was not significant in most measurements (Table 2), absolute differences between right and left M1 velocity of proximal segments were significantly larger in TCD than in 4D flow MRI (EDV, 7.21 cm/s vs. 2.96 cm/s, respectively, *p* = 0.003; MV, 8.89 cm/s vs. 4.05 cm/s, respectively, *p* = 0.002).

## 4. Discussion

Intracranial velocity measurements using TCD and non-contrast 4D flow MRI demonstrated strong correlations in the bilateral M1 segment, and the correlation between the two modalities increased from the distal to proximal segments. Mean velocities acquired with non-contrast 4D flow MRI were approximately 8 to 10% lower than those acquired by using TCD according to the location of M1. We believe that non-contrast 4D flow MRI is a reliable technique that enables comprehensive hemodynamic assessment as well as flow velocity quantification.

Previous studies using a phase-contrast technique demonstrated that phase-contrast MRI underestimates known velocity values acquired from Doppler ultrasound [7,22,24,25,26], which is in line with our result. We also observed that PSV had a larger measurement difference between the two modalities than EDV, which is thought to be owing to the temporal averaging effect of 4D flow MRI. TCD has real-time temporal resolution, which captures the PSV of a single heartbeat, while 4D flow MRI has a lower temporal resolution than TCD [7,22]. If the PSV varies from heartbeat to heartbeat, averaging velocity over multiple cardiac cycles during an MRI scan may affect the maximum velocity [22]. Chang et al. [22] reported that mean velocities measured using contrast-enhanced 4D flow MRI are approximately 30% lower than those acquired by using TCD.

On large vessels, the application of contrast has no significant effect on the reconstructed velocity profile [27,28]; however, the situation is quite different for small vessels such as intracranial arteries [23]. Lagerstrand et al. compared pre- and post-contrast phase-contrast MRI for intracranial flow velocity measurement and revealed that the use of a contrast agent resulted in underestimating the maximum velocity [23]. Our study found a smaller gap in velocity measurement between 4D flow MRI and TCD than in the contrast-enhanced 4D flow MRI study [22]. We speculate that the use of a contrast agent induces a more stationary signal, which contributes to pixel values. Thus, complex signal averaging results in a lower pixel value at the point of the highest flow velocity [23]. Therefore, we believe the non-contrast technique is a requisite for 4D flow MRI for the purpose of intracranial velocity measurements.

In addition to not using contrast agents, our study differs from the previously published studies [7,22], which compared 4D flow MRI and TCD for intracranial velocity measurement in some respects. We achieved significantly shorter acquisition time (10–12 min) with higher spatiotemporal resolution compared to Meckel et al. (25–30 min) [7]. We performed a subsegmental analysis of the M1, and we observed a high correlation between the two modalities, especially at the proximal segment. The correlation between the two modalities increased from the distal to the proximal segments. We attribute this result to the blind nature of TCD; because the most common variations in the anatomical bifurcation are located in the mid to distal segment, it is not easy to measure the same location as was measured using TCD. Lastly, as this was a prospective study, the time interval between TCD and 4D flow MRI was very short (0.7 ± 1.6 days), which could minimize bias owing to hemodynamic variations.

Vasospasm, which is known to be related to the inflammatory response caused by subarachnoid hemorrhage, is a critical condition that may cause confusion or focal neurologic deficit [29,30]. TCD plays a pivotal role in daily bedside monitoring of patients with subarachnoid hemorrhage for detecting vasospasm [2]. Although 4D flow MRI has weaknesses in terms of feasibility in daily bedside monitoring, it allows to assess various hemodynamic parameters such as flow rate, total volume in the whole brain vessels, including distal cerebral arteries, which are otherwise difficult or impossible to assess using TCD alone. Future outlook using 4D flow MRI will be to predict symptomatic vasospasm after researching the relationship between vascular perfusion and regional tissue perfusion. Furthermore, 4D flow MRI can be performed with the non-contrast technique; thus, it has advantages over CT angiography, which is also widely used to evaluate vasospasm after subarachnoid hemorrhage [31].

In our study, five patients were deemed unsuitable for MCA flow assessment using TCD owing to poor temporal windows (three bilateral and two unilateral). By contrast, 4D flow MRI is free from the limitations of insufficient temporal bone windows and allows the retrospective analysis of regions that were previously not expected to be relevant [13]. Moreover, intracranial 4D flow MRI has revealed good test–retest reliability, multicenter reproducibility, and interobserver agreement [32].

Intracranial 4D flow MRI has a promising role in investigating cerebrovascular pathologies. Futami et al. identified aneurysm vortex cores on 4D flow MRI and found an association between a complex vortical flow pattern and complex aneurysm morphology [20]. Thus, they concluded that identification of the vortex core flow patterns using 4D flow MRI may help in stratifying the aneurysm rupture risk. In a study with patients who underwent extracranial-intracranial bypass, 4D flow MRI provided multiple hemodynamic information such as flow direction, bypass patency, and blood volume data for each artery of interest [33]. Clinical feasibility of 4D flow MRI may be enhanced by addressing the primary technical issue of long scan time. Emerging MRI techniques including undersampling in both spatial and temporal domains as well as multi-velocity encoding acquisition may expand the application of 4D flow MRI in stroke patients [34]. Further study using 4D flow MRI is expected to provide hemodynamic clues for the mechanisms of post-ischemic hyperperfusion after intravascular treatment.

One of the study limitations is that we did not set a large field of view of the 4D flow MRI to include the transforaminal window level. To achieve clinically feasible scan times, our scan covered the area from the mid V4 segments of the vertebral artery to the A3 of the anterior cerebral artery. This might substantially impede our ability to find the exact TCD measurement location when using the distance correlation method. We speculate that this limitation may cause a weak correlation between TCD and 4D flow MRI at the basilar artery. However, the unknown relationship of the insonated artery to the scan plane [35], an inevitable drawback of TCD, may also contribute to the weak correlation between TCD and 4D flow MRI at the basilar artery. Second, a higher spatial resolution could be needed for the small-sized intracranial vessels to reduce partial volume artefact of the phase information. Since hemodynamic parameters such as mean flow velocity, peak flow velocity, and wall shear stress are influenced by spatiotemporal resolution [14,36], we expect that higher resolution with a short scan time can be achieved with seven tesla MRI.

## 5. Conclusions

Our study revealed that non-contrast 4D flow MRI and TCD correlated well for intracranial artery velocity measurements. Further, 4D flow MRI is a powerful tool for both radiologists and clinicians, enabling simultaneous quantitative flow velocity measurement and providing an unprecedented ability of 3D blood flow visualization. With further developments in the MRI technique, we expect that 4D flow MRI would be clinically feasible with short scan time and play a promising role in the analysis of cerebrovascular disease.

## Figures and Tables

**Table 1:** Mean of peak systolic velocity, end-diastolic velocity, and mean velocity measured by transcranial Doppler ultrasound and four-dimensional flow MRI.

			TCD (cm/s)	4D Flow MRI (cm/s)	Correlation
Right	Distal M1	PSV	66.22	57.29	0.49 *
		EDV	33.22	32.69	0.53 *
		MV	45.45	41.22	0.57 *
	Mid M1	PSV	76.27	70.06	0.68 *
		EDV	38.95	38.27	0.73 *
		MV	54.40	49.53	0.74 *
	Proximal M1	PSV	77.36	73.10	0.69 *
		EDV	37.59	40.98	0.73 *
		MV	53.59	51.69	0.72 *
Left	Distal M1	PSV	67.35	61.54	0.39
		EDV	32.85	33.05	0.48 *
		MV	46.75	42.55	0.44 *
	Mid M1	PSV	80.40	72.39	0.61 *
		EDV	40.60	40.15	0.60 *
		MV	56.20	50.89	0.58 *
	Proximal M1	PSV	82.57	74.54	0.67 *
		EDV	41.68	40.12	0.81 *
		MV	58.05	51.59	0.78 *
Basilar artery		PSV	56.18	57.23	0.17
		EDV	26.86	32.68	0.57 *
		MV	38.90	40.86	0.41

* Significant difference at 0.05 level. Abbreviations: 4D, four-dimensional; EDV, end-diastolic velocity; MV, mean velocity; PSV, peak systolic velocity; TCD, transcranial Doppler.

**Table 2:** Absolute values of mean difference between right and left M1 velocities.

		TCD (cm/s)	4D Flow MRI (cm/s)	*p*-Value
Distal	ΔPSV	11.21	11.86	0.890
	ΔEDV	5.84	5.87	0.981
	ΔMV	7.68	7.69	0.994
Mid	ΔPSV	10.05	12.09	0.470
	ΔEDV	5.05	4.04	0.435
	ΔMV	7.21	6.46	0.654
Proximal	ΔPSV	10.94	7.69	0.124
	ΔEDV	7.21	2.96	0.003
	ΔMV	8.89	4.05	0.002

Abbreviations: ΔEDV, change in end-diastolic velocity; ΔMV, change in mean velocity; ΔPSV, change in peak systolic velocity; TCD, transcranial Doppler.

## Data Availability

Not applicable.

## Supplementary Materials

The following are available online at https://www.mdpi.com/article/10.3390/diagnostics12010023/s1, Video S1 (**a**) Color-coded three-dimensional (3D) rendering shows velocity vectors, which visualize the direction of blood flow; (**b**) Color-coded 3D rendering of flow velocity, which displays velocity changes according to the cardiac cycle; (**c**) Color-coded 3D rendering with streamlines, which represent the path of particles if the particles are released into the velocity field while the field is kept constant.
